# Exploring factors influencing the quality of life in diabetes: a network analysis-based study

**DOI:** 10.3389/fpsyt.2024.1431772

**Published:** 2024-11-18

**Authors:** Lu Chen, Ling Li, Kexin Qiao, Zhengxue Qiao, Ying Xiang, Jiawei Zhou, Tianyi Bu, Xiaomeng Hu, Siyuan Ke, Yuecui Kan, Xuan Liu, Yanping Ji, Xiaohui Qiu, Yanjie Yang

**Affiliations:** ^1^ Department of Health Prevention and Care, Beijing Hospital, Beijing, China; ^2^ Institute of Geriatric Medicine, Chinese Academy of Medical Sciences, Beijing, China; ^3^ Psychology and Health Management Center, Harbin Medical University, Harbin, China; ^4^ Department of Endocrinology, Second Affiliated Hospital of Harbin Medical University, Harbin, China; ^5^ 1 Department of Infectious Disease, the Fourth Affiliated Hospital of Harbin Medical University, Harbin, China

**Keywords:** network analysis, diabetes, quality of life, depression, mental health, medical coping

## Abstract

**Objective:**

The purpose of this study was to explore the key pathways leading to low quality of life in type 2 diabetes patients by means of network analysis, so as to provide the possibility of effective interventions.

**Methods:**

The study involved 1,011 adult type 2 diabetes patients from a tertiary hospital in Harbin. Data was collected through questionnaires, and network analysis was performed using R software to assess the centrality and predictability of each node.

**Results:**

“Depression” and “Submission” (weight = 0.26), “Depression” and “Physiological field” (weight = -0.16), exhibit the strongest associations. Overall, “Depression” has the highest weight in the association with diabetes symptom, regarding betweenness, “Depression” and “Submission” exhibit the highest scores, Furthermore, the analysis of closeness centrality reveals that “Depression” and “Submission” share the highest level of proximity, it suggests that they have the shortest distances to other network factors in our research network.

**Conclusion:**

Depression and Submission are likely to be key factors affecting the quality of life of people with diabetes. Providing psychological support and scientific coping strategies for diabetes patients may be an effective way to help them live a better life.

## 1 Introduction

**Figure 1:**
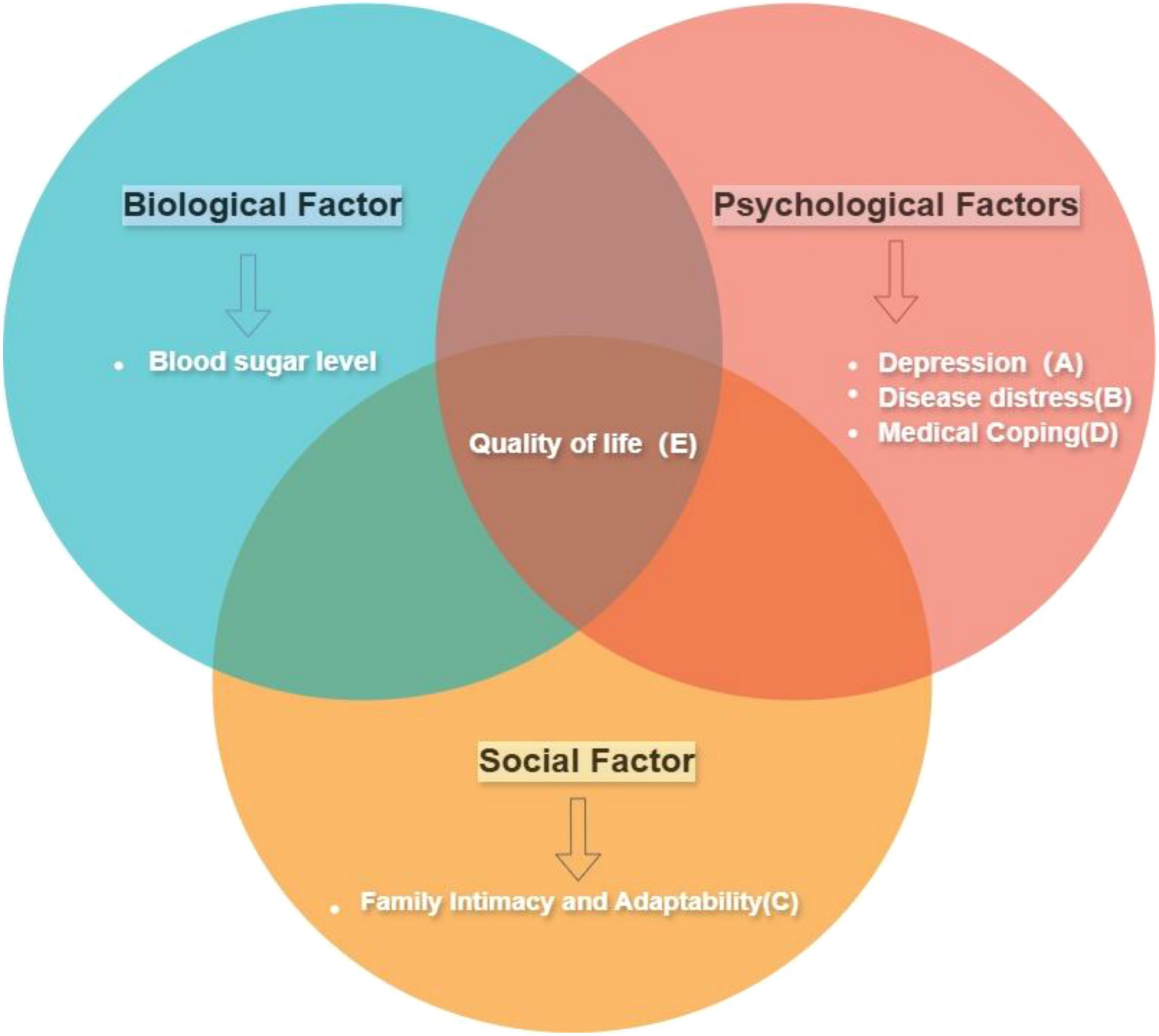
Conceptual framework diagram. This study utilizes the Bio-Psycho-Social Model framework, incorporating the variables shown in the diagram, to discuss the impact of these factors on the quality of life. The tags (A–C, etc.) correspond to the variables in the frame and will be used throughout the article: Depression(A), Disease Distress(B), Family Intimacy and Adaptability(C), Medical Coping(D), and Quality of Life(E). (While the study uses this framework, it focuses on psychological and social factors and quality of life, not including biological factors.).

Type 2 diabetes mellitus (T2DM) is a common and serious chronic disease that poses a growing threat to global public health (1). Diabetes is one of the leading causes of sickness, death, and decreased quality of life globally (2). The quality of life (QOL) is a critical component in patients’ experience of illness. It is evaluated based on physical and social functioning, as well as perceived physical and mental well-being. Numerous demographic and psychosocial factors influence quality of life. Improved quality of life (QoL) and appropriate diabetes self-management practices are crucial to improving and sustaining the health of diabetic patients. By giving more weight to the subjective evaluation of patients and examining various dimensions of life quality such as physiological, psychological, social relationships, and environmental field, we can develop a more holistic comprehension of patients’ encounters with the disease and treatment. This approach enables the establishment of personalized treatment objectives, the delivery of specific supportive measures and management strategies, thereby resulting in enhancements in the overall experiences and quality of life for patients (**Figure 1**).

According to the Bio-Psycho-Social Model, the interaction between Biological, psychological, and social factors frequently plays a role in the development of diseases, significantly impacting the quality of life for individuals with diabetes (3). Experiencing emotional distress related to diabetes is commonly witnessed among individuals with this condition (4). Each patient has a unique disease experience that encompasses emotional and cognitive aspects, ultimately shaping their coping strategies (5). Extensive research has indicated that coping strategies play a pivotal role in blood sugar control and overall health outcomes for individuals living with diabetes (6). In addition, the quality of life for individuals with diabetes is determined not only by personal factors but also by social support from sources such as family (7).

Contemporary research, which mainly emphasizes cross-sectional studies using methods such as regression analysis, mediation, and moderating effect analysis, often proves cumbersome and inefficient in elucidating the interrelationships between multiple variables in a single study. However, as demonstrated by numerous studies, the multifaceted nature of psychological phenomena emphasizes the intricate interplay of factors that contribute to their occurrence and evolution (8). Network analysis is a recent development in the field of psychology, by studying the topological structure of dynamic interactions between symptoms (9).It can deeply investigate the complex relationship between various variables, so as to identify the key variables within the network. This opens up the possibility of a comprehensive understanding of the quality of life in people with diabetes.

### 1.1 Study aim

The present study was to determine the relationship between the quality of life of diabetic patients and family factors and negative emotions through the method of network analysis, so as to identify the key pathway contributing to the low quality of life in patients with type 2 diabetes. The findings of this study are expected to help people with diabetes improve their mental state and restore their quality of life.

## 2 Methods

### 2.1 Participants

#### 2.1.1 Data sources

Participants in this study were hospitalized patients from a tertiary hospital in Harbin, Heilongjiang Province, China, and data were collected from the end of 2022 to the middle of 2023. Data on basic characteristics, behavioral lifestyles, psychological traits, and other relevant information were collected through questionnaires, while body measurements and blood glucose levels were obtained through physical examinations. This study adhered strictly to the principle of voluntariness. Prior to the survey, the purpose and significance of the research were explained to the participants, and they all voluntarily agreed to participate by signing informed consent forms. The privacy information and collected data of the participants were treated with utmost confidentiality, and no information about the participants was disclosed.

#### 2.1.2 Inclusion and exclusion criteria

The inclusion criteria for the study were: (1). Diagnostic criteria: fasting blood glucose ≥126 mg/dL (7.0 mmol/L) or glycated hemoglobin (HbA1c) ≥6.5%. (2). Age: The study included patients aged between 18 and 80 years. (3). The duration of the disease in these patients exceeded one year.

The exclusion criteria are: (1). Other conditions: The patient does not have any other known conditions that affect blood sugar control or cause major complications, such as cardiovascular disease, kidney disease, liver disease, etc. (2). Other studies: The patient is not participating in any other ongoing clinical trials or intervention studies that may have an impact on the patient’s blood sugar levels or other research outcomes. (3). Refusal to participate: The patient himself refused to participate in the study.

#### 2.1.3 Final sample size

The collected questionnaires and examination data were carefully verified, resulting in the exclusion of 640 individuals (32%) due to incomplete information regarding basic characteristics, psychological traits, and blood glucose levels, among others. Ultimately, a total of 1011 participants were included in the study. Of the participants surveyed, 596 (59.0%) were male and 415 (41.0%) were female (**Table 1**).

**Table 1:** Univariate analysis of quality of survival in diabetic patients.

Variable	N	%	Quality of life (M ± SD)	*F/t*	P
Gender				2.87	0.32
Men	596	59.0	54.45 ± 8.34		
Women	415	41.0	52.86 ± 8.99		
Marital status				0.37	0.77
Married	875	86.5	53.77 ± 8.56		
Unmarried	65	6.4	54.75 ± 9.04		
Divorced/Separated	22	2.2	53.84 ± 11.21		
Widowed	49	4.8	53.08 ± 8.53		
Residence				0.86	0.42
Cities	569	56.3	54.11 ± 8.69		
Counties	300	29.7	53.32 ± 8.55		
Villages	142	14.0	53.80 ± 8.64		
Education level				0.18	0.94
Middle school	110	10.9	53.66 ± 9.03		
Secondary	298	29.5	53.53 ± 8.57		
High school	307	30.4	53.84 ± 8.87		
College	278	27.5	54.02 ± 8.38		
Master	18	1.8	54.81 ± 8.47		

### 2.2 Measurements

Based on the existing literature, this study examines various factors, including individual demographic features, cultural characteristics, Depression(A), Disease Distress(B), Family Intimacy and Adaptability(C), Medical Coping(D), and Quality of Life(E) (**Table 2**).

**Table 2:** Descriptive statistics of psychological variables.

Variable	N	Minimum	Maximum	Mean	SD
Depression (A)	1011	25.0	85.0	52.956	10.205
Emotional burden (B1)	1011	1.0	6.0	2.192	0.998
Doctor-related pain (B2)	1011	1.00	6.00	1.638	0.872
Treatment program pain (B3)	1011	1.0	6.0	2.150	0.911
Interpersonal pain (B4)	1011	1.0	6.0	1.674	0.893
Family intimacy (C1)	1011	31	92	67.02	8.621
Family adaptive (C2)	1011	17	70	48.21	8.612
Confrontation(D1)	1011	10	32	20.00	3.240
Avoidance (D2)	1011	7	26	15.34	2.888
Submission(D3)	1011	5	18	9.91	2.944
Physiological field (E1)	1011	-9.71	19.42	13.19	2.11
Psychological field(E2)	1011	6.66	18.667	13.323	2.129
Social relations field (E3)	1011	4.0	20.0	13.682	2.991
Environmental field(E4)	1011	4.0	20.0	13.599	2.649

#### 2.2.1 Depression

Depression was assessed using Zung’s Self-Rating Depression Scale

(SDS). This scale consists of 20 items reflecting subjective feelings of depression. Each item is rated on a four-point scale based on the frequency of symptom occurrence, with 10 items positively scored and 10 items negatively scored. Using a four-point scoring method (1-4 points), the scores for the 20 items are summed to obtain the raw score. After applying a formula, the raw score is multiplied by 1.25, and the resulting value is rounded to the nearest integer to obtain the standard score for depression (10).

#### 2.2.2 Disease distress

The Diabetes Distress Scale (DSS) was utilized in this study to assess the level of diabetes-related strain experienced by patients. (11), this scale consists of four subcategories that capture different aspects of diabetes-related distress, including Emotional Burden(B1)(5 items), doctor-related distress (4 items), treatment-related distress (5 items), and interpersonal relationship distress (3 items). Participants responded to each question on a 6-point Likert scale, with options ranging from 1 (no problem) to 6 (extremely severe problem). The total score on the scale ranged from 17 to 102 (12).

#### 2.2.3 Medical coping

The Medical Coping Modes Questionnaire (MCMQ), developed by Feifel et al., is composed of three dimensions: Confrontation(D1), Avoidance(D2), and Submission(D3). The questionnaire consists of a total of 20 items and employs a 4-point Likert scale. The total score on the MCMQ ranges from 20 to 80, with eight of the items being reverse scored. A higher score indicates a greater tendency for patients to adopt a specific coping mode (13).

#### 2.2.4 Family intimacy and adaptability

The Family Adaptability and Cohesion Evaluation Scale (FACES-II), developed by Olson et al., was employed to assess emotional connections and adaptability among family members. The scale includes two dimensions: intimacy and adaptability. It consists of 30 items, rated on a 5-point scale (1 = “never” to 5 = “always”). A higher score on the scale signifies a greater level of family Intimacy and Adaptability(C) (14).

#### 2.2.5 Quality of life

The WHOQOL-BREF is a questionnaire widely used as a generic measure of quality of life. The WHOQOL-BREF contains 26 items which evaluate the four dimensions of quality of life (“Physical Health”, “Psychological Health”, “Social Relationships”, “Environment”) The scale is scored on a 1-5 scale, with certain items being reverse-scored. The total score ranges from 0 to 100, with higher scores indicating enhanced functioning within each dimension and an overall higher quality of life (15).

### 2.3 Data processing and statistical analysis

Network analysis was performed using R version 4.3.2. Initially, the graphical LASSO method combined with EBIC model selection was employed to estimate the network model and calculate weights for all potential connections. This step was automatically executed using the “estimate Network” function with the default options of “EBICglasso”. The network visualization represented the four symptoms of Quality of Life(E), four types of Disease Distress(B), Family Intimacy and Adaptability(C) for two symptoms, and three types of Medical Coping(D) as circles, referred to as “nodes”. The graph displayed labels, colors, and descriptions for these 14 items. Subsequently, the nodes were connected by “edges” represented by lines. The thickness of the edges indicated the strength (weight) of the connections between these nodes, while the color of the edges indicated the relationship, with blue symbolizing positive and red symbolizing negative connections.

The present study calculates the strength, betweenness, and closeness centrality indices for each node. Strength represents the node with the most robust overall connections. Betweenness based on its frequency of occurrence in the shortest paths between other nodes. The closeness centrality index gauges the average distance between a node and other nodes in the network, with smaller values indicating closer proximity. These indices offer insights into the relative influence of nodes within the network structure (16).

In order to examine the connections between Quality of Life(E) and different factors that impact it, we employed bridge analysis to identify key pathways. Bridge nodes are nodes that potentially act as links between larger node communities (17). In this study, we utilized the “bridge” function from the “network tools” package to calculate bridge strength and identify potential bridge nodes. Bridge strength is determined as the sum of the absolute values of all edges connecting a node with other nodes that are not part of the same community or structure. To ensure the reliability of our findings, we assessed the stability of the network, which reflects the robustness of the estimates generated by the model and should be evaluated prior to interpreting the network. To test the accuracy of edge weights and the stability of centrality, we employed the “bootnet” package in R, which incorporates a case-dropping function. We conducted a nonparametric bootstrapping method (n = 2000) to create multiple plausible datasets from the observed data, to assess the “edge weight accuracy.” “Centrality stability” is measured by the Centrality Stability (CS) coefficient for centrality indices (e.g., strength), which indicates the maximum number of cases that can be dropped to ensure a 95% probability that the correlation between the original centrality index and the centrality in the network based on data subsets is 0.50 or higher. It is recommended that CS be at least above 0.25, preferably higher than 0.50, for the interpretation of centrality measures. In our network, all CS values are above 0.5, demonstrating the reliability of the network.

## 3 Result

**Figure 2:**
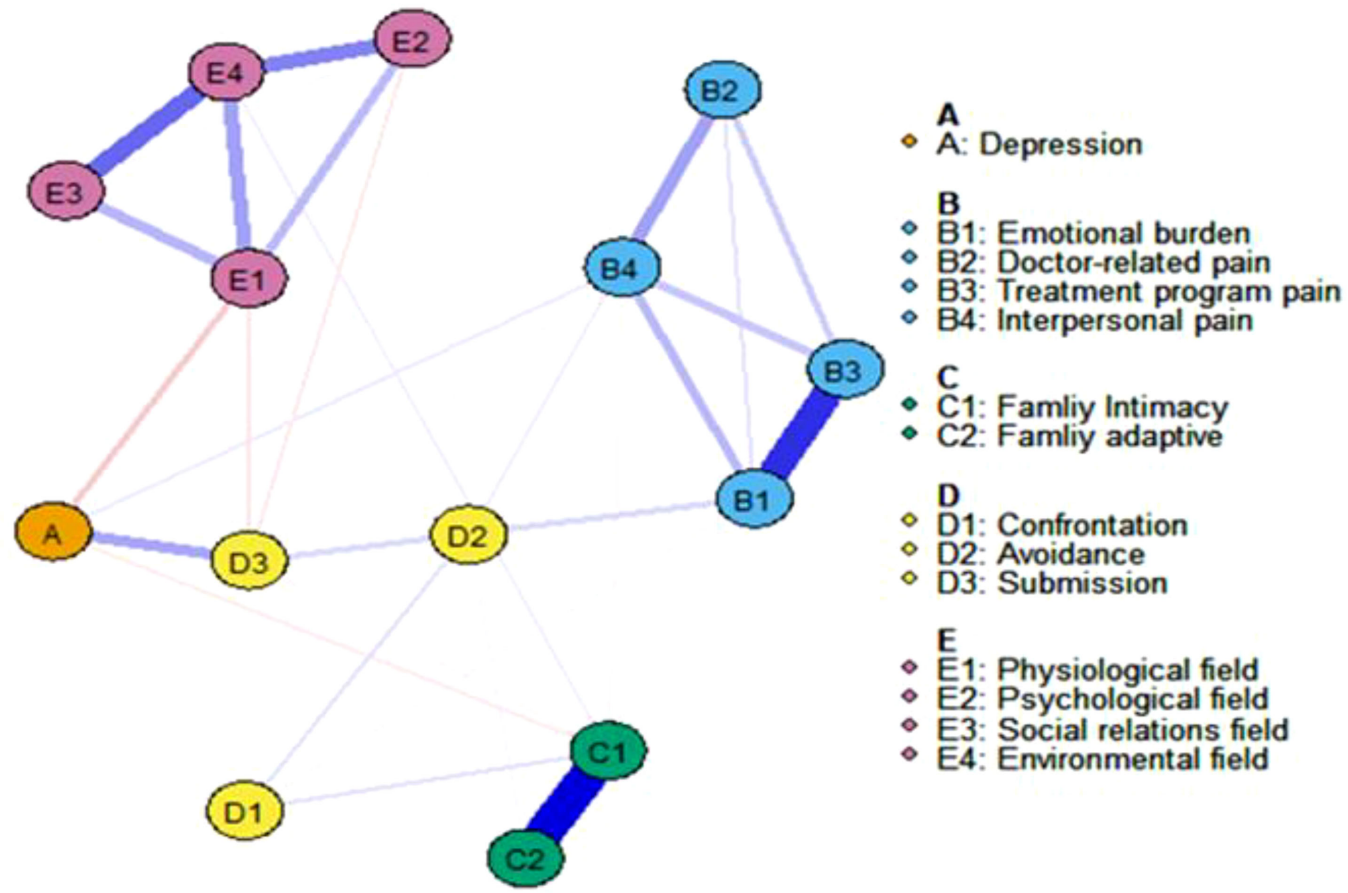
The network displaying the relationship about all items. Blue edges represent positive correlations between variables; red edges represent negative correlations. The thickness of an edge represents the absolute magnitude of the correlation. Different colored nodes were defined as different communities.

**Figure 2** illustrates the estimated network of social-psychological risk factors and diabetes burden. The weighted associations of edges connecting these 14 nodes in the network are shown in the table. Out of 91 possible edges, 57 edges (54.28%) have non-zero weights. We identified the strongest associations between five different social-psychological factors and diabetes burden. We found that “Depression(A)” and “Submission(D3)” (weight = 0.26), “Depression(A)” and “Physiological field(E1)” (weight = -0.16), “Emotional Burden(B1)” and “Submission(D3)” (weight = 0.12), “Family Intimacy(C1)” and “Confrontation(D1)” (weight = 0.10), and “Submission(D3)” and “Physiological field(E1)” (weight = 0.09) exhibit the strongest associations. Overall, Depression(A) has the highest weight in the association with diabetes, indicating its significant impact on diabetes.

**Figure 3:**
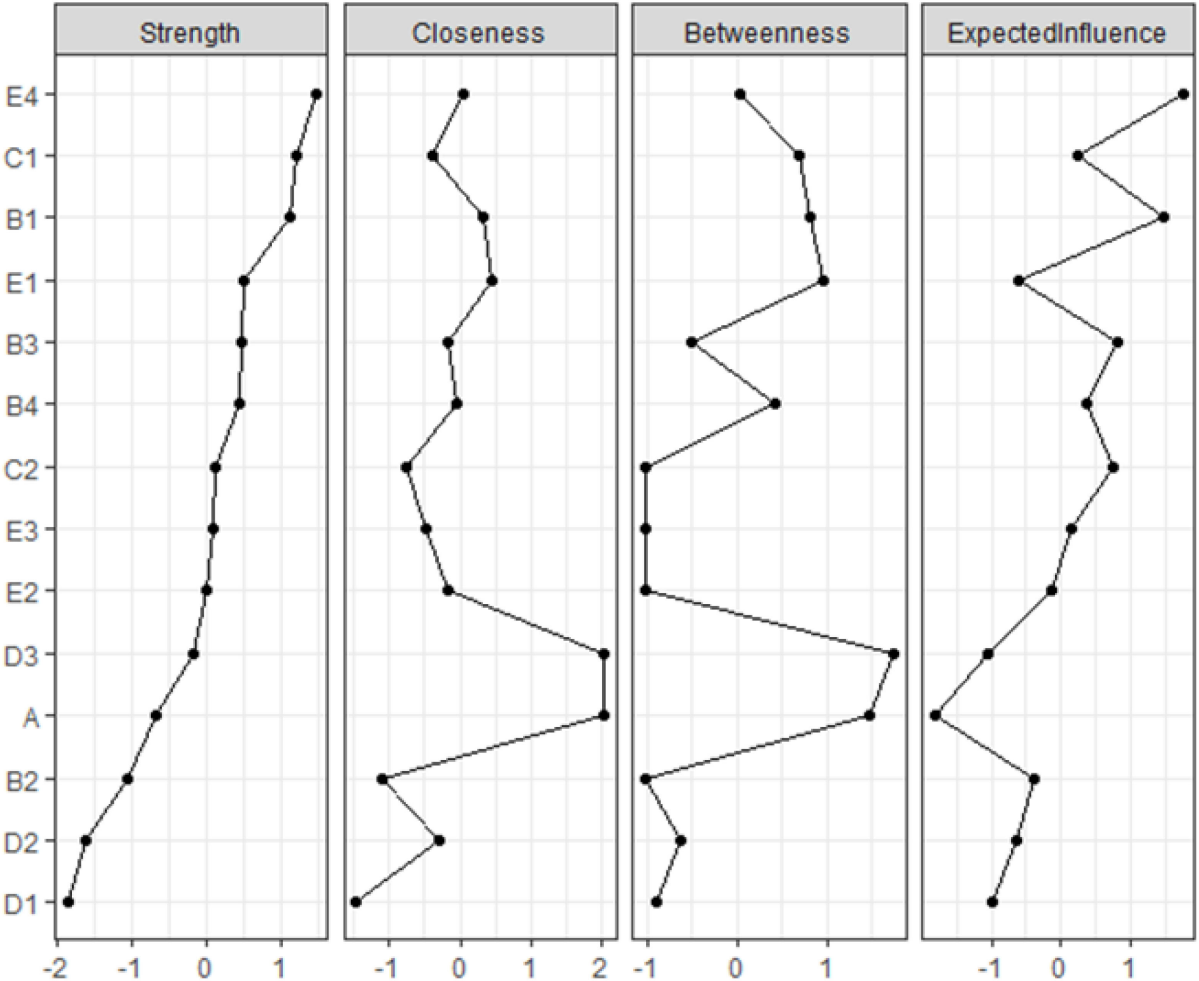
The centrality estimates for each node in the network. Presented strength, betweenness, and closeness centrality indices are standardized (i.e., z-scores).

The network’s node centrality characteristics, namely strength, betweenness, and closeness, are illustrated in **Figure 3**. Strength-wise, the nodes demonstrating the highest correlation with symptoms or risk factors are “Environmental Field(E4),” “Family Intimacy(C1),” “Emotional Burden(B1),” and “Physiological Field(E1).” These findings indicate that these items are deeply intertwined with other elements in the network. Regarding betweenness, “Depression(A)” and “Submission(D3)” exhibit the highest scores. Furthermore, the analysis of closeness centrality reveals that, in addition to intra-variable relationships, Depression (A) and Submission (D3) have the shortest average distance to all other nodes in the network. This indicates that both variables are centrally located within the network, facilitating quicker access to and interaction with other variables.

**Figure 4:**
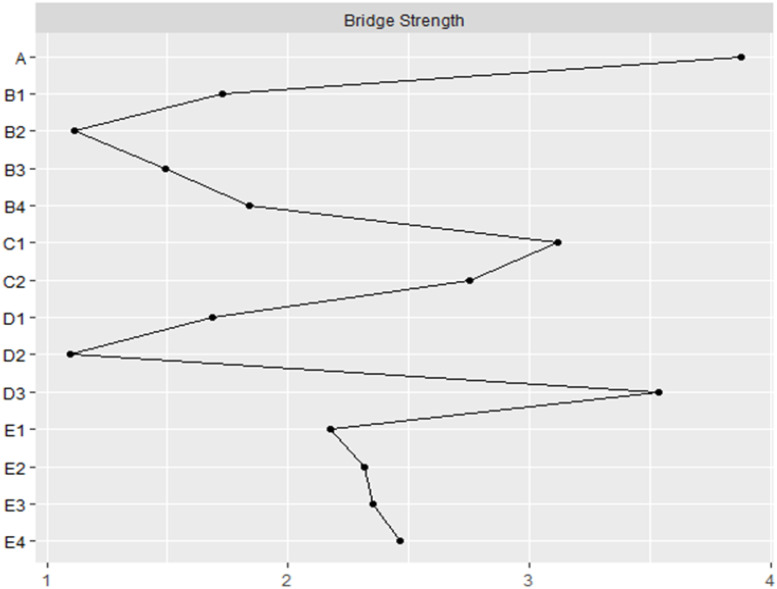
The bridge strength (z-score) of each variable chosen in the final network.

To gain a deeper understanding of the connections between five communities, the bridge strength of each node was computed (see **Figure 4**). Higher values of symptom/environment measures indicate a stronger significance in linking nodes from one community to another, excluding nodes within the same community. Depression (A) emerges as the highest bridge factor, indicating that it plays a crucial bridging role in the network of factors influencing the quality of life in diabetes patients. It demonstrates strong connections with the B-C-D-E group of factors.

**Figure 5:**
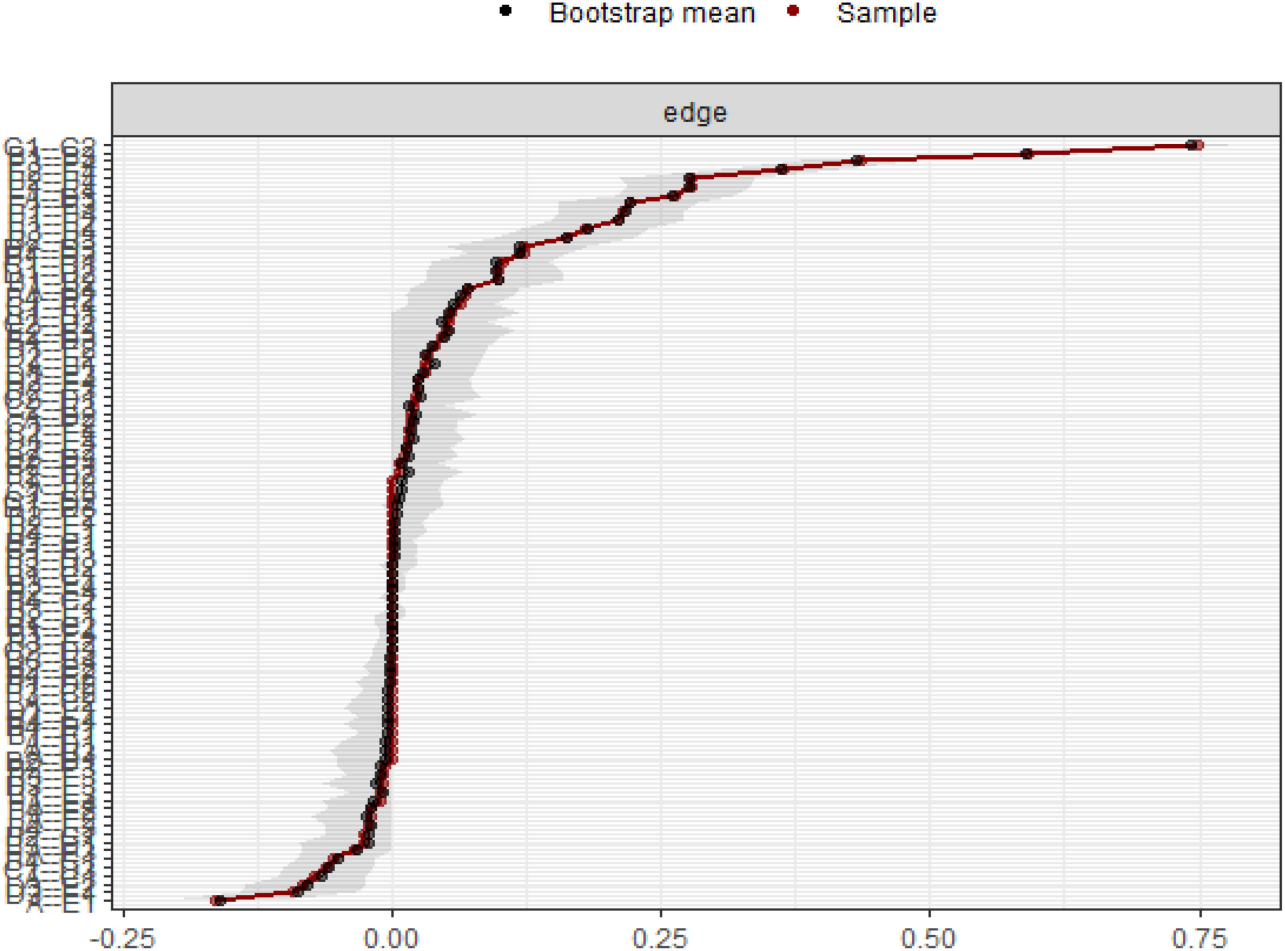
Bootstrap 95% confidence intervals of the edge weight.

**Figure 6:**
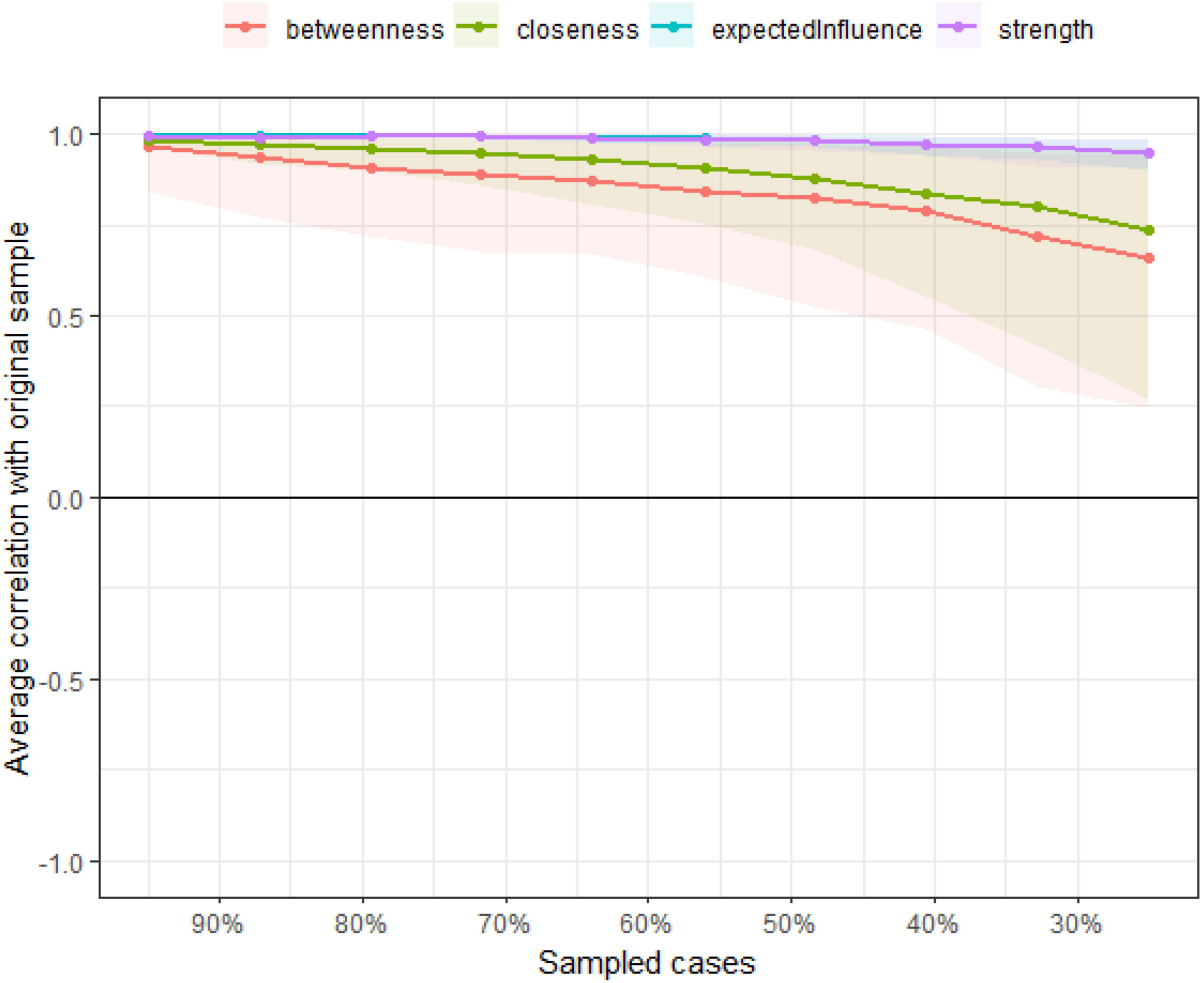
The case-dropping subset bootstrap procedure demonstrates.

Using the case-drop bootstrap method to calculate confidence intervals, the 95% confidence intervals for edge weights are relatively narrow and exhibit significant overlap (see **Figure 5**). This indicates a high level of accuracy in estimating the network, with many notable differences between the intervals. The case-drop subset bootstrap procedure further demonstrates the stability of betweenness, closeness, and strength values even after removing a significant portion of samples (**Figure 6**). Both betweenness and closeness metrics display similar levels of stability, with a CS-C value of 0.361. In contrast, the strength index in this sample proves to be highly robust and reliable, with a CS-C value of 0.75. As described in the Methods section, a coefficient lower than 0.25 is not recommended, and an ideal value exceeds 0.5. Thus, the network exhibits a certain level of stability.

## 4 Discussion

This study employs a network analysis approach to investigate the relationships among Depression(A), Disease Distress(B), Family Closeness and Adaptability(C), Medical Coping(D), and Quality of Life (E) in a diabetic population. Traditional analyses often focus on specific factors in isolation, relying on preconceived assumptions about their interactions and correlations. In contrast, network analysis provides a comprehensive view of how these factors interrelate, facilitating a more exploratory analysis of multivariate influences on disease outcomes.

In the context of diabetes, Medical Coping (D) emerges as a key determinant of quality of life. Family support, alongside Depression (A) and Disease Distress (B), plays a crucial role in shaping patients’ attitudes toward managing their illness. Within the network, each factor not only independently influences quality of life but also interacts with others in complex, bidirectional ways. Thus, improving quality of life necessitates a broader perspective that encompasses multiple dimensions, including physiological, psychological, and familial factors.

### 4.1 Depression and quality of life in diabetes

Within this cluster, Depression(A) emerges as the symptom most strongly associated with Quality of Life(E). Depression in diabetic patients is frequently chronic and persistent, posing obstacles in health management and potentially leading to disease progression and a deterioration in Quality of Life. Furthermore, the relationship between Depression and diabetes is bidirectional; the presence of both conditions can exacerbate the Quality of Life, impair blood sugar control, decrease medication adherence, heighten complications, and increase the risk of suicide (18). This study’s findings diverge from previous research by emphasizing that Depression (A) not only negatively impacts the Psychological Field (E2) but also has a profound effect on the Physiological Field (E1) of Quality of Life (E). Contributing factors include suboptimal blood sugar regulation, heightened complication risks, compromised functional abilities, increased healthcare costs, and elevated mortality rates (19). Consequently, healthcare professionals should focus on recognizing and addressing the physiological and psychological changes in diabetic patients. Timely interventions, including psychological support and education, can help alleviate emotional burdens, enhance coping abilities, and ultimately improve quality of life.

### 4.2 The relationship between remaining variables and quality of life

This study reveals that several variables, beyond Depression (A), significantly influence the quality of life in diabetes patients. Specifically, Submission (D3), as part of Medical Coping (D), mediates the impact of Depression on overall quality of life. Research indicates that negative coping strategies, such as submission, are correlated with heightened levels of Depression (20), which can exacerbate the Emotional Burden (B1) faced by individuals managing diabetes. Moreover, diabetes management often entails strict adherence to treatment protocols, which can lead to increased emotional strain. Patients may struggle with self-criticism (21) and find it challenging to accept their condition, complicating their coping strategies (22). This emotional turmoil can lead to feelings of weakness and despair, resulting in a surrendering attitude that diminishes motivation for effective treatment.

In addition to these psychological factors, family dynamics play a critical role in influencing coping strategies. The study finds a significant correlation between family intimacy and the coping strategy of “confrontation” in medical contexts. Existing literature supports this by demonstrating that a supportive family emotional environment positively impacts patients’physiological systems and enhances self-care behaviors (23). Practical support from family and friends is essential for encouraging adherence to medication, diet, and exercise, thereby achieving optimal blood sugar control and reducing the risk of long-term complications. Overall, the interplay between these variables underscores the importance of addressing both psychological and familial factors in enhancing the quality of life for diabetes patients.

### 4.3 Depression treatment and family support for better quality of life

The most immediate improvement in quality of life arises from the treatment of depression. Unfortunately, in many low- and middle-income countries, diabetes care lacks comprehensiveness, with inadequate processes for identifying and addressing psychological and psychiatric issues. To bridge this gap, healthcare providers must prioritize both mental health and medical outcomes. By offering interventions such as psychological support, psychoeducation, and psychotherapy, patients can alleviate their emotional burdens and enhance their coping abilities.

In conclusion, recognizing family dynamics and coping styles as modifiable risk factors is crucial for improving quality of life. It is essential to help families feel valued and enhance their sense of control. Family support is vital not only for biomedical management (including metabolism, care, nutrition, and complications) but also for improving quality of life and reducing the stress associated with chronic illnesses. Meeting patients’ emotional needs is fundamental. This insight calls for a reevaluation of public health strategies, emphasizing the role of family support in shifting patients’ attitudes toward their illness and alleviating psychological distress, ultimately improving quality of life.

## 5 Strengths and limitations

The key innovation of this study is the application of network analysis to explore the complex relationships between psychosocial factors and quality of life in diabetes patients. Unlike traditional analytical methods, network analysis delves deeper into the dynamic interactions between these factors, emphasizing both direct and indirect pathways that influence patient health. This approach provides valuable insights for clinicians, helping them develop multi-dimensional interventions to improve patients’ quality of life across various aspects, rather than focusing solely on individual symptoms.

Despite offering a novel perspective, this study has certain limitations. Firstly, as a cross-sectional design, it does not allow for causal inference. Future research could employ longitudinal designs, performing cross-lagged panel network (CLPN) analysis, which would enable the dynamic exploration of the complex relationships between symptoms proposed by the network model in longitudinal data. Additionally, this study primarily focuses on psychological and social factors, while the role of physiological factors, especially in a metabolic disease like diabetes, should not be overlooked. Future research should integrate physiological indicators (e.g., glycated hemoglobin) to offer a more holistic perspective for diabetes management.

## 6 Conclusion

This study utilizes network analysis to explore the relationships among Depression(A), Disease Distress(B), Family Intimacy and Adaptability(C), Medical Coping(D), and Quality of Life(E) in diabetes patients. Findings show that Depression(A) and Submission(D3) significantly impact both psychological and physical well-being, while family support promotes positive coping strategies like Confrontation (D1). These results highlight the need for integrating mental health interventions and family support in diabetes care. Healthcare services should prioritize addressing psychological issues, enhancing family involvement, and implementing comprehensive support strategies.

## Data Availability

The original contributions presented in the study are included in the article/supplementary material. Further inquiries can be directed to the corresponding authors.
